# Effect of fecal microbiota transplant on symptoms of psychiatric disorders: a systematic review

**DOI:** 10.1186/s12888-020-02654-5

**Published:** 2020-06-15

**Authors:** Arthi Chinna Meyyappan, Evan Forth, Caroline J. K. Wallace, Roumen Milev

**Affiliations:** 1 Department of Psychiatry, Queen’s University, 752 King St. West, Kingston, ON K7L 4X3 Canada; 2 Providence Care Hospital, 752 King St. West, Kingston, ON K7L 4X3 Canada; 3 Centre for Neuroscience Studies, Queen’s University, 18 Stuart St., Kingston, ON K7L 3N6 Canada; 4 Department of Psychology, Queen’s University, 62 Arch St., Kingston, K7L 3L3 ON Canada

**Keywords:** Fecal microbiota transplant, Gut-brain axis, Psychiatric illness, Depression, Anxiety, Eating disorders, Substance abuse, Chronic stress

## Abstract

**Background:**

The Gut-Brain-Axis is a bidirectional signaling pathway between the gastrointestinal (GI) tract and the brain. The hundreds of trillions of microorganisms populating the gastrointestinal tract are thought to modulate this connection, and have far reaching effects on the immune system, central and autonomic nervous systems, and GI functioning. These interactions Diagnostic and statistical manual of mental disorders have also been linked to various psychiatric illnesses such as depression, anxiety, substance abuse, autism spectrum disorder, and eating disorders. It is hypothesized that techniques aimed at strengthening and repopulating the gut microbiome, such as Fecal Microbiota Transplant (FMT), may be useful in the prevention and treatment of psychiatric illnesses.

**Methods:**

A systematic search of five databases was conducted using key terms related to FMT and psychiatric illnesses. All results were then evaluated based on specific eligibility criteria.

**Results:**

Twenty-one studies met the eligibility criteria and were analysed for reported changes in mood and behavioural measures indicative of psychiatric wellbeing. The studies included were either entirely clinical (*n* = 8), preclinical with human donors (*n* = 9), or entirely preclinical (*n* = 11). All studies found a decrease in depressive and anxiety-like symptoms and behaviours resulting from the transplantation of healthy microbiota. The inverse was also found, with the transmission of depressive and anxiety-like symptoms and behaviours resulting from the transplantation of microbiota from psychiatrically ill donors to healthy recipients.

**Conclusion:**

There appears to be strong evidence for the treatment and transmission of psychiatric illnesses through FMT. Further research with larger sample sizes and stronger scientific design is warranted in order to fully determine the efficacy and safety of this potential treatment. Registered on PROSPERO, IRD: CRD42019126795.

## Background

In recent years, there has been a growing appreciation for research in the field of the “gut-brain axis” (GBA). The GBA consists of bidirectional biochemical and neural signalling between the gastrointestinal (GI) tract and the brain. Specifically, the gut microbiota is able to modulate the GBA both directly and indirectly via endocrine, neural, and immune pathways. In disease- or stress-states these pathways may become compromised resulting in intestinal dysbiosis, changes in mood, behavior, and cognition, and altered inflammatory levels [1].

The gastrointestinal tract is colonized by over one hundred trillion commensal bacteria that exist symbiotically with our bodies. Colonization of the gut occurs at birth and is largely influenced by mode of delivery (c-section vs. vaginal birth) and through breast feeding. However, bacterial composition of the gut begins to stabilize throughout adulthood [2]. Detailed analyses of human gut microbiota have shown considerable individual variability in bacterial content as this dynamic system is influenced by a variety of factors, such as genetics, diet, metabolism, age, geography, antibiotic treatment, psychotropics, and stress [3, 4].

Gut microbiota are critical in the normal development of the immune system, central nervous system (CNS) circuitry, GI functioning, and autonomic nervous system (ANS) functioning. This community of bacteria and its genetic material is often referred to as a virtual organ [4–6]. Studies have since shown that gut bacteria play a vital role in regulating important aspects of brain development and function, along with other host physiology [5, 7].

### The gut and psychiatric symptoms and disorders

The interaction of the gut with the environmental risk factors of psychiatric illnesses, such as diet and early life stress, suggests that interventions targeting the gut microbiome could prevent and treat psychiatric symptoms [8]. Psychiatric symptoms can manifest in both psychological and physiological ways, often resulting in impaired functioning. Common physiological symptoms share similarities with symptoms of GI disorders, such as Irritable Bowel Syndrome (IBS). This association may be explained by the close connection between the gut and the brain.

In past studies, individuals with psychiatric illness have also been shown to have a dissimilar microbiota composition compared to healthy individuals, due to decreased diversity and abundance of the healthy gut microbes [9]. Studies also show that lack of exposure to commensal bacteria, such as in germ-free mice, has significant effects on stress responsiveness in adulthood; it has also been shown that early colonization of the gut with a conventional microbiota, even a single species, can partially reverse these effects [10]. Some investigations have shown neurochemical changes as a result of gut microbiome dysfunction, such as altered levels of brain-derived neurotropic factor (BDNF), reduced serotonin receptor expression, reduced synaptic plasticity gene expression, and increased striatal monoamine turnover [10–12].

### Fecal microbiota transplant

Several methods of examining the influence of the gut microbiome on the gut-brain axis have been explored, including manipulating the microbiome via probiotic and antibiotic administration, the use of germ-free animal models, and perhaps most notably, fecal microbiota transplantation (FMT). FMT is the transfer of fecal bacteria from a healthy donor to a recipient. FMT was first used in fourth century China for the treatment of severe food poisoning and diarrhea and other related symptoms [13]. However, it is currently only indicated for the treatment of *Clostridium difficile (C. difficile.)* infections. *C. difficile* is often contracted by older patients in-hospital following routine pharmacological treatments such as antibiotics. The use of antibiotics often depletes healthy bacteria in the GI tract which can result in microbial dysfunction. FMT is used to restore healthy status of the microbiome via repopulation of healthy bacteria to the gut. Functioning in a similar manner to probiotics, this treatment method helps to maintain the bacterial balance and function. FMT are most commonly accomplished via endoscopies, enemas, and oral feeding of freeze-dried material. Aside from GI and psychiatric disorders, this treatment method is also being explored as a potential treatment for metabolic disorders, autism, multiple sclerosis, and Parkinson’s disease [14–17]. Other variations of this treatment, such as Microbial Ecosystem Therapeutics-2 (MET-2) are also currently being explored, in psychiatric indications such as Generalized Anxiety (GAD) and Major Depressive Disorders (MDD). MET-2 consists of gut bacteria obtained from stool samples of a healthy donor, chosen for its safety profile, that is then purified and lab-grown prior to being lyophilized and ingested orally by patients [18].

### FMT in the context of psychiatric illness

Two of the most prevalent groups of psychiatric disorders include Major Depressive Disorder and anxiety disorders. MDD is characterized by either depressed mood and/or loss of interest or pleasure, as well other psychiatric and physiological symptoms. Anxiety disorders is a category that includes a variety of disorders characterized by intense feelings of anxiety, nervousness, or fear. These include Generalized Anxiety Disorder, Agoraphobia, Panic Disorder, and specific phobias. Both groups of disorders are characterized by a significant impairment in daily functioning [19]. While there are pharmacological treatments available for both disorders, many people deny treatment due to side effects or stigma-related reasons or are treatment-resistant and unable to find an effective way to improve their symptoms. By targeting the gut, FMT may be a potential way to overcome these drawbacks. Research on the gut-brain axis indicates that there may be a possibility to improve these symptoms through restoration of the gut microbiome via fecal transplant from a healthy donor. However, as this is a relatively novel area of research, there are few studies on FMT in humans as a treatment method in the context of psychiatric disorders.

This review examines findings from preclinical and clinical studies that have examined the effects of endogenous microbiome transfer on psychiatric symptoms. The studies included in this review assess the effects of FMT and related interventions on symptoms associated with a variety of psychiatric illnesses including MDD, anxiety, and chronic stress. Comorbid disorders associated with poor mental health outcomes such as alcoholism and anorexia were also included in several of the studies.

## Methods

### Literature search strategy

**Fig. 1:**
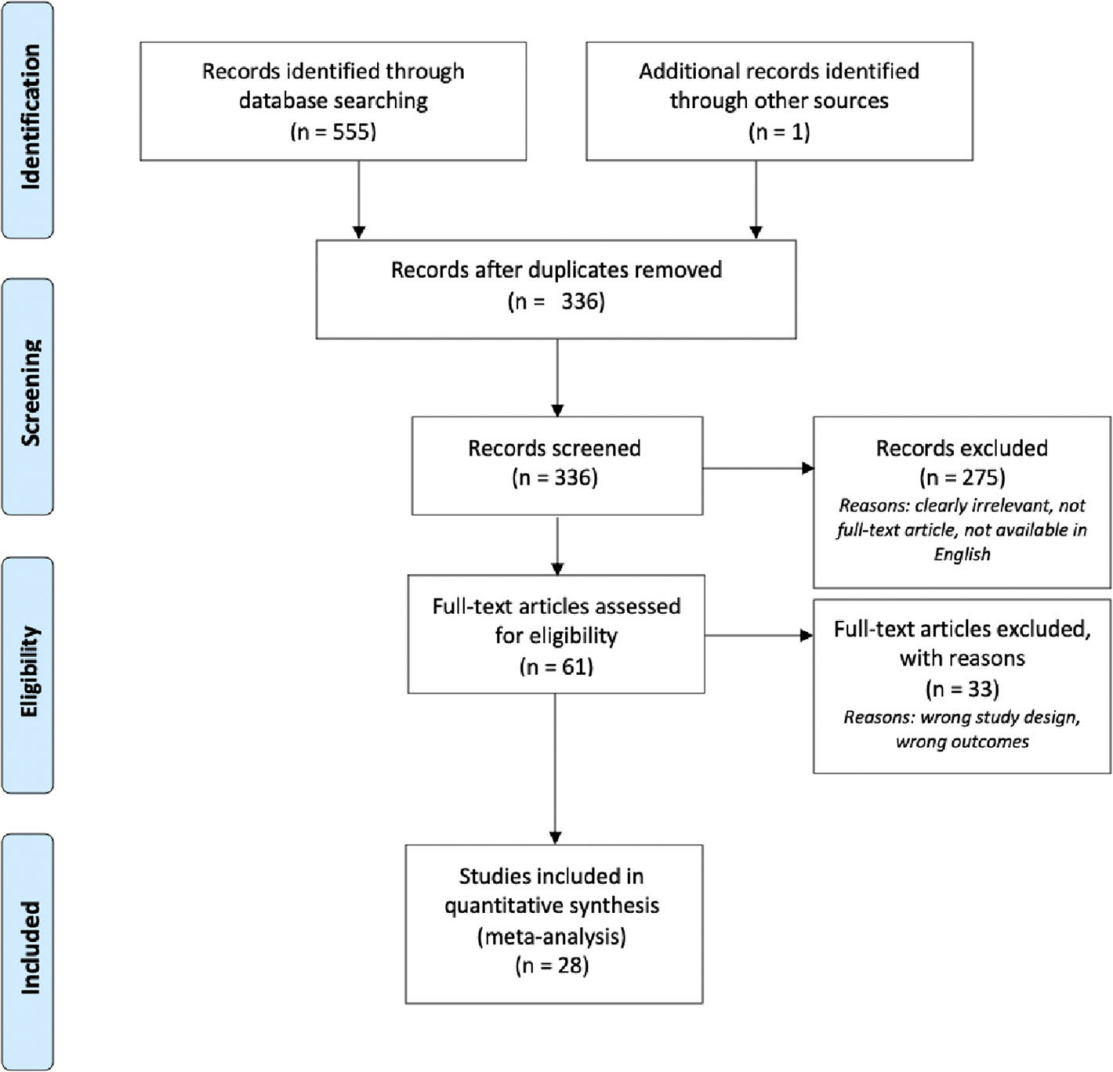
Flow chart showing literature search and screening process using PRISMA process

This review adhered to the Preferred Reporting Items for Systematic Reviews and Meta-Analyses (PRISMA) guidelines (Fig. 1, [20]). Relevant studies were identified by systematically searching the following databases: MEDLINE, EMBASE, CINAHL, PsycINFO, and Web of Science using key search terms including: mood, anxiety, mania, stress, phobia, microbiota transfer, and fecal transplant. The strategy adapted to each of the databases listed above and is described in detail in Appendix 1. Searches were conducted in November 2019 and yielded 285 studies after duplicates were removed. The search was updated in April 2020, yielding 7 new studies after full-text screening. Any studies that were excluded during full-text screening were due to wrong study design or outcomes. Reasons for which articles were rejected on the basis of ‘wrong study design’ include the article being a review paper or only an abstract. Those that were rejected due to “wrong study outcome” did not measure any clinical symptoms directly related to psychiatric illness.

### Eligibility criteria

All articles eligible for inclusion were published in peer-reviewed journals and were written in English. The studies were restricted to preclinical or clinical samples that were assessed for changes in symptoms of psychiatric illness after undergoing an endogenous microbe transfer via any route of administration.

### Study selection

One author (A.C.M) completed initial search of the databases, adhering to the search strategy (Appendix 1). Two authors (A.C.M and C.W) independently assessed the titles and abstracts of records retrieved from the systematic search according to the identified inclusion and exclusion criteria and completed the full-text review. Any disagreements were resolved by a third author (R.M).

### Study quality

The Cochrane Handbook for Systematic Reviews of Interventions Risk of Bias Tool (RoBT) addresses 6 specific domains in assessing the quality of randomized controlled trials: sequence generation, allocation concealment, blinding, incomplete outcome data, selective outcome reporting and ‘other issues’. All included studies were analyzed according to these domains by A.C.M and C.W. As there is limited research on this topic, studies that did not follow the double-blind, randomized controlled model, were included if they had a comprehensive methodology after thorough analysis by three of the authors (A.C.M., C.W., and R.M.). Overall, the included studies presented with low levels of potential bias save for cases where blinding of either the participant or study team was not performed. For these cases, bias was assessed as high. Detailed assessments can be found in Table 1. Allocation concealment was not assessed since no studies concealed treatment allocation from subjects or participants.

## Results

### Study characteristics

This review contains 28 studies evaluating the effect of fecal microbiota transplants on various psychiatric and physical symptoms. Of these, eleven studies examined exclusively animal samples, nine studies were preclinical subjects with transplanted microbiota from human donors, and eight studies examined exclusively human samples. The characteristics of these studies are displayed in Tables 2, 3, and 4. The average sample size was *n* = 23 for entirely preclinical studies, and *n* = 78 for preclinical studies with human donors, and *n* = 19 for clinical studies. Four of the preclinical studies failed to report rodent sample size. The symptoms most frequently studied were that of Irritable Bowel Syndrome (IBS), chronic stress, and depressive-like symptoms.

### Preclinical studies

In the eleven preclinical studies included in this review (Table 2), the primary indications investigated were chronic stress (*n* = 6), alcoholism (*n* = 2), chronic neuropathic pain (*n* = 2), and depression (*n* = 1). Of the studies assessing chronic stress, three investigated the effects of FMT from mice subjected to the chronic unpredictable mild stress (CUMS) procedure to healthy mice, one replicated this using the social defeat model of stress in rats, one investigated the effects of fecal transplant from healthy mice to stressed mice, and one investigated the effects of FMT from NLPR3 knockout mice on the resiliency of mice subjected to the CUMS procedure after fecal transplant. When investigating the effects of FMT from CUMS mice to germ free mice, three studies found that the FMT resulted in increased anxiety and depression-like behaviour [21–23]. Mice were assessed using the open-field, tail suspension, forced swimming, and elevated plus maze tests – all standard tests for evaluating symptoms related to stress and depression in the field of depression research in mice. Pearson-Leary et al. conducted a similar study, by using the social defeat rodent model of stress. Male rodents were introduced into cages with retired male breeder rats that were preselected for aggression. This creates a situation where the experimental rats are subjected to several episodes of social defeat by the older, aggressive rats, leading to chronic stress. These rats were then identified as SL/vulnerable (exhibiting passive behaviours and short-latencies to defeat) or LL/resilient (exhibiting active coping behaviours and long-latencies to defeat). SL/vulnerable rats displayed increased depressive and anxiety-like behaviours, whereas LL/resilient rats did not. Naïve rats then received FMT from SL/vulnerable, LL/resilient, or control rats. It was found that rats receiving FMT from the SL/vulnerable group displayed depressive-like behaviours when asses with the forced swim test while the LL/resilient group was not significantly different than control [24].

Langgartner et al. investigated the alleviating properties of FMT by designing a study in which mice were split into chronic subordinate colony housing (CSC) and non-stressful single housing (SHC). CSC housing is a validated way to subject mice to chronic psychological stress, similar to the CUMS procedure. The researchers then evaluated if frequent FMT from SHC to CSC mice could stop the development of anxiety and depression-like symptoms. It was found that these transplants were mildly stress protective, resulting in decreased anxiety and depression-like symptoms in the recipient mice [25]. Zhang et al. also investigated the protective properties of FMT by transplanting the microbiota from NLPR3 knockout mice into GF mice. NLPR3 is a gene involved in the aforementioned immune pathway that modulates the GBA. The transcripts of this gene have been found to be increased in both patients with depression and mouse models for depression [26, 27]. Knocking out this gene is thought to be protective against the development of depressive-like symptoms in mice. These mice were then subjected to the CUMS procedure in order to see the effects of the FMT on their resiliency. It was found that FMT from NLPR3 knockout mice resulted in a decrease in anxiety and depressive-like behaviours in the recipient mice [28].

Xiao et al. evaluated the transfer of alcohol-withdrawal symptoms via FMT. In this study, the donor mice were treated with alcohol – they were forced to ingest alcohol for 2 weeks, with the concentration of alcohol increasing from 5 to 35% over that time period. The fecal microbiota of these mice was then transplanted into healthy control mice. This transplantation resulted in depressive behaviour in the recipient mice. This behaviour was evaluated using the forced swim and tail suspension tests [29]. Jiang et al. conducted a similar study treating mice with alcohol and transplanting their microbiota into germ free mice, but some alcohol treated mice were treated with nicotinamide riboside afterwards. Similar results were found after FMT from alcohol treated mice, but treating the donors with nicotinamide riboside before transplantation stopped the transmission of depressive-like behaviours [30]. Additionally, Tillmann et al. also studied the use of FMT to transfer depressive behaviour, using a Flinders sensitive line (FSL) and Flinders resistant line (FRL) rats. FSL and FRL rats have been widely used for over 30 years as a depression model in rats [31], where FSL rats display depressive-like behaviours, and FRL rats are resistant to the development of depressive-like behaviours. Tillman et al. Investigated the effects of FMT from FSL and FRL rats to FSL, FRL and saline control groups. The only significant behavioural results found were an increased immobility in the forced swim test and decreased time spent in the centre of the open field test resulting from FMT from FSL to control rats, and that rats receiving FRL feces struggled less in the forced swim test than those receiving saline [32] . The former suggests the transfer of depression symptoms from FSL rats to others, and the latter provides evidence against the transference of resiliency from FRL rats. Yang et al. measured pain and depression symptoms in mice after FMT from rats with neuropathic pain presenting with and without anhedonia. Rats were administered a spinal neuropathic injury (SNI) and then assessed for anhedonia susceptibility. After being separated into anhedonia-susceptible and resilient groups, the rats’ microbiota was then transplanted into mice, and the mice were assessed for pain responses and depression-like symptoms. It was found that transplants from anhedonia susceptible rats aggravated pain and depression-like symptoms, and those receiving from anhedonia resilient microbiotas had improved pain and depression-like symptoms. The reverse was conducted by Schmidt et al. 2020, investigating the the effects of FMT from healthy rats to rats given a spinal cord injury (SCI) [33]. It was found that the transplantation of a health microbiota resulted in a reduction of anxiety and depressive-like symptoms in mice subjected to SCI, as measured by the elevated plus maze and light-dark tests.

### Preclinical studies with human donors

Of the nine preclinical studies performing FMT from human donors to germ free (GF) mice (Table 3), four assessed the effects of FMT from patients with depression, one from patients with alcoholism, one from patients with anorexia and one from patients with IBS. Two studies assessed the ameliorating properties with the transfer of gut microbiome from healthy human controls to mouse models of alcoholism and autism spectrum disorder respectively. These mice were then assessed for changes in various tests used to indicate change in psychiatric state.

After FMT from individuals with depression to GF mice, three studies found a decrease in centre motion in the open field test [34–36]. Two studies also found a significant increase in immobility duration in the tail suspension and forced swim tests [35, 36]. Liu et al. found increase in immobility duration in the forced swim test only [37]. Although Kelly et al. did not find any changes in the forced swim test, they found a decrease in the total visits to the open arms in the elevated plus maze [34]. These four studies all support that FMT from patients with depression to GF mice can result in depression-like behaviour in the recipient mice.

Alcoholism is highly comorbid with mood and anxiety disorders, with someone with alcoholism being 3.6 and 2.6 times more likely to have a mood or anxiety disorder respectively [38]. In the studies investigating the effect of FMT on depressive and anxiety-like behaviors related to alcoholism, Zhao et al. found that transplants from patients with alcoholism to GF mice resulted in depression and anxiety-like behaviours in the aforementioned tests [39]. The inverse was conducted by Xu et al. – in their study, mice were treated with alcohol and as a result these mice displayed depression and anxiety-like symptoms in the same tests. FMT was then performed from healthy human donors at different time points throughout the alcohol treatment. Xu et al. found that when administering 3 FMTs per week, if the transplants began before or during the alcohol treatment, the depression and anxiety-like behaviours were not observed [40]. Chen et al. conducted a similar study investigating the positive effects of FMT from healthy humans to mice models for autism spectrum disorder. The mice either received a transplant from the original pooled human gut microbiome, or from cultured bacteria from the original pooled human microbiome. It was found that both the in vitro cultured and non-cultured bacteria transplants resulted in anxiety-like behaviours assessed through the marble burying and self-grooming tests, but that only the non-cultured bacteria transplants resulted improved performance during the open field test.

Anorexia is also closely tied to mood and anxiety disorders, with the likelihood of someone with anorexia having a comorbid mood or anxiety disorder being 2.4 and 1.9 times higher respectively than the general public [41]. When assessing the effects of FMT from patients with anorexia to germ free mice, Hata et al. observed an increase in compulsive and anxiety-like behaviours in the mice when assessed using the open field and marble burying test. As previously seen, the mice spent significantly less time in the centre of the open field test, suggesting anxiety-like behaviour. The mice also buried more marbles than control mice, suggesting compulsive behaviour. The mice also buried more marbles than control mice, suggesting compulsive behaviour [42]. These findings are also consistent in De Palma et al.’s study where transfer of anxiety and IBS symptoms to GF mice via FMT was assessed. In their study, it was found that transplants from anxious IBS donors resulted in more anxiety-like behaviour in recipient mice. This was not the same for transplants from IBS donors without anxiety [43]. These studies all show that FMT can confer certain traits of the donor’s psychiatric illnesses to the recipient mouse, and that transplants from healthy donors may be able to alleviate some psychiatric symptom.

### Clinical studies

In contrast to the preclinical studies with human donors, where transplants were primarily from ill and healthy humans into GF mice, in clinical studies (Table 4), the fecal microbiota of healthy volunteers were transplanted into humans with illnesses such as IBS and depression. All eight clinical studies assessed for psychiatric symptoms – six studies assessed depressive symptoms, four assessed anxiety symptoms, one assessed neuroticism, two assessed quality of life in relation to IBS, and one assessed fatigue. Depression symptoms were assessed in four of the six clinical studies using the Hamilton Depression Rating Scale (HAM-D). Other scales used to assess depression symptoms were the Patient Health Questionnaire (PHQ-9), the Quick Inventory of Depressive Symptomatology (QIDS) and the Hospital Anxiety and Depression Scale, depressive sub-scale (HADS-D). All of these studies found a significant short-term improvement in depression symptoms. The long-term effects were less consistent, with three studies finding a return to baseline at week 12, week 20, and month 6 respectively [44–46]. Xie et al., however, found a persistent decrease in depression symptoms lasting up to 17 months after the final round of FMT [47].

Of the four studies assessing anxiety symptoms, three used the Hamilton Anxiety Rating Scale (HAM-A), and one used the Hospital Anxiety and Depression Scale, anxiety sub-scale (HADS-A). Three of the four studies found a significant improvement in anxiety symptoms following FMT [44, 45, 48]. Although Mizuno et al. found improvement in the anxiety scores as well, it was not significant [44]. As with depression symptoms, Huang et al. found the anxiety scores to return to baseline within 6 months post-transplant and Mazzawi et al. found the improvement to be insignificant by week 20 (Huang et al. 2019 [45]; Mazzawi et al. 2018 [46]).

Neuroticism was assessed using the Eynsek Personality Questionnaire-Neuroticism (EPQ-N-12) by Mazzawi et al. A significant decrease in EPQ-N-12 scores was seen at week 3, but this returned to baseline by week 20 [46]. Huang et al. and Johnsen et al. assessed quality of life using the IBS-QOL scale. Scores on this questionnaire followed a similar pattern as the assessments above, with a significant improvement being observed during the first 3 to 6 months and returning to baseline after 6 to12 months [45, 49]. Johnsen et al. also found a similar effect on fatigue, with improvement up to 6 months after FMT and a waning effect from 6 to 12 months [49].

## Discussion

The findings from reviewing the included studies suggest that FMT can affect symptoms of psychiatric disorders. This was shown for both the relief of psychiatric symptoms resulting from the transfer of microbiota from healthy donors to ill recipients and the transmission of symptoms through the transplantation of microbiota from ill donors to healthy recipients. This relationship was investigated in a variety of psychiatric disorders including depression, anxiety, anorexia and alcoholism. The transmissible properties of FMT were also well demonstrated in these studies. Notably, regardless of donor species, the transmission of psychiatric symptoms from ill donors to GF mice was consistently found. This was supported in multiple studies, with observed transference of symptoms from mouse models of depression, anxiety, chronic stress and alcoholism, and from humans with depression, to GF mice. This provides support for the concept that the gut microbiome may both contribute to the development of psychiatric disorders and be a viable target for treatment for these disorders.

All included clinical studies found improvement in the symptoms of psychiatric disorders related to these disorders following FMT from healthy donors. The beneficial aspect of FMT from healthy donors was also demonstrated preclinically where healthy transplants resulted in alleviation of depression- and anxiety-like symptoms that appeared in mice subjected to certain conditions. This alleviation of symptoms was found in mice experiencing alcohol withdrawal, as well as stressful living conditions. Though symptom alleviation was consistently observed, the duration of improvement was inconsistent. Some studies, such as Xie et al., observed an alleviation of symptoms that seemed to last indefinitely, but the majority found this to be transient (Xie et al. 2019 [45]; H. L. Huang et al. 2019 [46]; Mazzawi et al. 2018 [44]; Mizuno et al. 2017 [47]). The benefits seemed to last for only around 3–6 months, which, if used as a treatment for psychiatric disorders, is a limitation for FMT in clinical practice.

### Mechanism of action

The mechanism of action for how this gut microbiome modulation results in the observed symptomatic changes has yet to be fully understood. There are currently a few major hypotheses for how the microbiome affects the nervous system, resulting in symptomatic changes. The papers included in this study discussed some of these theories, with the majority postulating the mechanism to be through changes in serotonin production, immune response, and metabolism in response to microbiome changes. Serotonin transmission has long been known to be altered in depression, with selective serotonin reuptake inhibitors (SSRIs) being the most prescribed treatment for depression [50]. An estimated 90% of the body’s serotonin is produced by enterochromaffin (EC) cells in the digestive tract [51]. The functioning of these cells has been known to be affected by gut microbiome changes. One way that microbiome disruption is thought to affect serotonin production is through short chain fatty acids (SCFAs). SCFAs are produced by the gut microbiome through the fermentation of non-digestible carbohydrates, suggesting that treatments that ameliorate gut health can influence SCFA concentrations. SCFAs, particularly butyrate and propionateinfluence the synthesis of the rate limiting enzyme tryptophan hydroxylase which synthesizes serotonin produced by EC cells [52, 53]. In addition to their role in serotonin production, SCFAs also have the ability to cross gut-blood and blood-brain barriers during immune signaling. The immune system can be affected by the gut simply by the fact that there are many immune cells located in the gastrointestinal tract, meaning that gut disruption can also disrupt these cells. The SCFAs produced by the gut microbiome have anti-inflammatory properties and can work to regulate the immune response [54]. In the gut, they influence expression of anti-inflammatory markers, such as interleukin (IL-)10 in macrophages and intestinal dendritic cells [55]. In the central nervous system, SCFAs have additional roles, such as regulating maturity and function of microglia (macrophages in the brain that are part of the brain’s immune response) [56]. Many psychiatric disorders have been linked to inflammation and an increased immune response, as observed through elevated levels of immune marker cytokines [57]. It is hypothesised that this response is mediated by the NLRP3 inflammasome, a multiprotein intracellular complex that activates pro-inflammatory cytokines [58].

A more direct way that the microbiota influences the central nervous system is through interaction with the vagus nerve. The vagus nerve is comprised of 80% afferent nerve fibers and 20% efferent fibers. Afferent nerve fibers of the vagus nerve are affected by metabolites of the microbiota that then take that information back to central nervous system [59]. This is hypothesized to influence central and peripheral changes resulting in alleviation of psychiatric symptoms. More specifically, the vagus nerve is affected by long and short chain fatty acids both directly and indirectly, through cellular production of neurotransmitters, such as serotonin [60].

These mechanisms may provide insight into the use of FMT as a potential treatment for psychiatric symptoms, such as mood and anxiety. The repopulation of the gut microbiome with healthy bacteria through FMT may have positive neurological, immune, and metabolic effects which in turn may influence the trajectory of the psychiatric indication.

### Treatment feasibility

Using FMT as a treatment for psychiatric illnesses in the future is an interesting idea that merits exploration. MDD and anxiety disorders affect millions of people worldwide and have a very large burden to the individual and society as a whole. The current gold-standard treatment for psychiatric illnesses, MDD and anxiety disorders in particular, are antidepressants medications. Though antidepressants have a relatively high efficacy, a large proportion of individuals with psychiatric illnesses do not respond to these first-line treatments, and thus need to try alternatives [61]. Further, many antidepressant users also experience side effects such as restlessness, nausea, vomiting, anxiety, insomnia, sexual dysfunction, gastrointestinal cramps and diarrhea, and headaches that can make the arduous process of searching for effective treatments even harder [62]. Antidepressant medications are also still steeped in stigma further impeding one’s ability to ask for and receive help and treatment. Finally, as is stands, on average antidepressants can be costly, especially without insurance or government-funded healthcare.

There is a great need for new therapeutic targets and treatments in order to provide options and better help individuals suffering from these psychiatric illnesses. When considering the findings demonstrated in this review, FMT appears to be a promising candidate for this. The ongoing research certainly suggests its efficacy and given the few side effects and adverse events reported in these papers, may even challenge the treatments currently available. Though the treatment effect seems transient, symptoms appeared to improve relatively quickly after treatment. Another common issue seen in these indications are often to do with treatment adherence. However, given FMT effects can last up to 6 months, it may be easier to adhere to than a daily medication or a weekly psychotherapy appointment. Assuming one transplant is sufficient for therapeutic benefits lasting up to 6 months, the cost of treatment may be comparable to that of brand-name antidepressants, however not much is known about the costs of FMT (Eisenberg Centre at Oregon Health & Sciences University, 2007). There is potential, however, for cost of FMT to decrease, as treatment becomes more mainstream and modified.

Though the effectiveness and tolerability of FMT, as seen in these studies, makes it a promising potential treatment, there are some aspects that could limit its adoption into mainstream clinical settings. A potential drawback currently is the procedure itself. Although costs are comparable to antidepressants, it is still relatively expensive and a labor-intensive alternative to other psychiatric treatments. Additionally, the safety of FMT has also not been sufficiently understood and its associated stigma is still unknown. These points, along with the treatment still being in the early stages of research, make it difficult to fully determine the feasibility of FMT as a treatment for psychiatric illnesses such as depression and anxiety.

### Limitations

Although the studies included in this review were of good quality and contributed to a greater understanding of FMT in the context of mental health and illness, there are considerable limitations. A significant limitation in any FMT study is the fact that although research on the gut microbiome has been prolific, we still do not know what a ‘healthy microbiome’ is. Some researchers refer to a healthy microbiome as one of an individual with no overt diseases and others, however, even among those who are considered healthy, the variation in taxonomic composition is great [63–65].

The main limitation of the clinical studies were small sample sizes. The lack of large-scale, double blind randomized controlled trails makes it difficult to determine efficacy and safety. The majority of clinical studies also assessed the psychiatric symptoms in individuals with IBS, and not necessarily those exclusively with psychiatric disorders. This means that, though there was clear improvement if psychiatric symptoms, it cannot conclusively be said that FMT will improve the symptoms of individuals with psychiatric disorders. Additionally, it is possible that the improvement of psychiatric symptoms is secondary to the improvement of gastrointestinal symptoms associated with IBS, thus is not a direct relationship.

For the preclinical studies using human donors, the sex of the mice and donors was a major limitation, given most studies used donors or mice of only male sex. Given that there are clear sex and gender differences in the prevalence and symptomatology of mental illnesses, further research is warranted to determine if sex and gender have an effect on the efficacy of FMT procedures. Some of these studies also included donors that were taking various medications, including antidepressants, which may have affected the results. Additionally, the administration of antibiotics to create GF recipient mice and the variability in FMT administration protocol make the findings difficult to translate. For instance, some recipients received multiple FMTs, while others received only one and the justification for choosing donors varied. The heterogeneity of indications studied also creates difficulty in knowing, with any certainty, how efficacious this procedure will be for a given indication. Without a consensus on a standard procedure for conducting this research, it is difficult to compare results between studies.

## Conclusion

With high individual variability in symptomatology and prognosis, high levels of comorbidity with other disorders, genetic *and* environmental influences, progress in research in treatment of psychiatric disorders has been challenging. Given the huge heterogeneity of psychiatric disorders, finding treatment that works for all patients is not achievable, especially given the range of factors that influence the disorder and treatment response. While the research in this field is far from complete, the potential of targeting the gut-brain axis using FMT to alleviate symptoms of psychiatric illness is promising. Additionally, given the adaptable nature of the gut microbiome, it may be a good representation of the individual’s history and could explain differences in risk of illness, disease course, and response to treatment. If these therapies are able to alleviate symptoms of psychiatric disorders, they could be offered to some patients as personalized, alternative, and/or adjunctive treatments to combat specific symptoms that tie together specific gut bacteria strains or the gut, as a whole, to the brain.

## Data Availability

All data analysed for and presented in this paper are from the twenty-one studies we reviewed. The data is accessible via referenced articles.

## Appendix

### Appendix 1. Search Strategy

1. Depression OR Mood OR Stress, Psychological OR Anxiety OR Mania OR Panic OR Phobia OR Psychiatric illness
2. Microbiota Transplant OR Stool transplant OR Bacteriotherapy OR Microbial Ecosystem Therapy OR Microbial/Microbe Therapy
3. 1 AND 2
  - MEDLINE: 206 Results
  - OVID PsycINFO: 15 Results
  - EMBASE, 206 Results
  - Web of Science, 111 Results
  - CINAHL: 1 Result

### Appendix 2

**Table 1:** Quality assessment details

Study	Blinding of outcome assessors for all outcomes		Blinding of participants and personnel for all outcomes		Incomplete outcome data for all outcomes	Selective outcome reporting	Sequence generation	
	Judgement	Comments	Judgement	Comments	Judgement	Judgement	Judgement	Comments
Cai 2019 [66]	High	Single outcome group, no blinding of any parties involved	High	Single case, no blinding of any parties involved.	Unclear	Unclear	High	Single case, no sequence generation
Tillmann 2019 [32]	Low				Low	Low		
Kurokawa 2018 [48]	High	Outcome assessors not blinded.	High	Participants and personnel not blinded.	Low	Low		
DePalma 2017 [43]	High	Investigators not blinded for behavioural and gastrointestinal motility tests.	Low		Low	Low	Low	
Xiao 2018 [29]	Low		Low		Low	Low	Low	
Mazzawi 2018 [46]	High	Outcomes assessors not blinded.	High	Participants and personnel not blinded.	Low	Low	Low	
Xu 2018 [40]	Low		Low		Low	Low	Unclear	
deClercq 2019 [67]	High		High		Unclear	Unclear	High	
Huang 2019 [36]	Low		Low		Low	Low	Unclear	
Huang 2019 [45]	High	Outcomes assessors not blinded.	High	Participants and personnel not blinded.	Low	Low		
Li 2019 [21]	Unclear		Unclear		Low	Low	Low	
Zhang 2019 [28]	Low		Low		Low	Low	Unclear	
Xie 2019 [47]	High	Outcome assessors not blinded.	High	Participant and personnel not blinded.	Low	Low		
Zhao 2019 [39]	Low		Low		Low	Low	Low	
Kelly 2016 [34]	Unclear		Unclear		Low	Low	Low	
Mizuno 2017 [44]	Low		High	Participants and personnel not blinded.	Low	Low		
Langgartner 2018 [25]	High	Outcome assessors, aside from histological scoring, were not blinded.	High	Personnel not blinded.	Low	Low	Low	
Yang 2019 [68]	Unclear		Unclear		Low	Low	Low	
Hata 2019 [42]	Unclear		Unclear		Low	Low	Unclear	
Lv 2019 [22]	Unclear		Unclear		Low	Low	Low	
Zheng 2016 [35]	Low		Low		Low	Low	Low	
Pearson-Leary 2019 [24]	Low		Low		Low	Low	Low	
Schmidt 2020 [33]	Low		Low		Low	Low	Low	
Johnsen 2020 [49]	Low		Low		Low	Low	Low	
Jiang 2020 [30]	High	Outcome assessors not blinded.	Low		Low	Low	Low	
Chen 2020 [69]	High	Outcome assessors not blinded.	Low		Low	Low	Low	
Siopi 2020 [23]	Low		Low		Low		Low	
Liu 2020 [37]	High	Outcome assessors not blinded.	Low		Low	Low	Low	

**Table 2:** Preclinical studies

Study	Sample characteristics	Study design	Intervention	Donor	Measurement	Key findings and conclusions
Zhang et al. 2019 [28]	Chronic Stress Mice	Randomized Controlled Trial	FMT from wildtype (WT) and NLRP3 KO mice to chronic unpredictable stress (CUS) mice	WT and NLRP3 KO mice	SPT, FST, TST, and OFT	• Transplantation of the NLRP3 KO gut microbiota ameliorated CUS-induced depressive-like behaviors.
Li et al. 2019 [21]	Antibiotic treated 8 male WT and 8 male chronic unpredictable mild stress (CUMS) mice	Randomized Controlled Trial	Oral FMT for 2 weeks from WT and CUMS mice to antibiotic-treated WT and CUMS mice	8 WT mice and 8 CUMS mice	SPT, OFT, EPM, FST	• FMT of CUMS microbiota induced anxiety-like and depression-like behavior in the recipient mice
Lv et al. 2019 [22]	Antibiotic treated Male rats with and without CUS	Randomized Controlled Trial	3-day oral FMT from WT and CUMS mice to antibiotic-treated rats with and without CUS	WT and CUMS mice	SPT, OFT, EPM, FST	• Transplantation of CUMS Microbiome Induces Depression-Like Behaviors in Antibiotic-Treated Rats as shown via a decrease in time spent in the central area in the OFT and increased immobility in the TST
Xiao et al. 2018 [29]	6–8 week male C57BL/6 mice	Randomized Controlled Trial	14 days of saline or oral FMT from alcohol or water exposed mice to healthy control mice	Alcohol-exposed and water-exposed mice	FST and TST	• FMT from alcohol-exposed mice induced depressive behavior in the recipients, shown by significant results in FST and TST
						• Alcohol withdrawal induced symptoms were transmitted to healthy controls
Yang et al. 2019 [68]	Antibiotic treated two-month-old male C57BL/6 mice	Randomized Controlled Trial	14 days of FMT from rats to antibiotic treated C57BL/6 mice	Two-month old	Mechanical withdrawal test (MWT), Tail flick test (TFT), SPT, locomotion, TST, and FST	• Antibiotic administration significantly aggravated the MWT scores, latency of TFT, and depression-like behaviors.
				Sprague Dawley rats with and without anhedonia		
						• FMT from rats with anhedonia significantly aggravated behavioral abnormalities, pain, depression-like, and anhedonia-like behaviors in recipient mice
						• Antibiotics-treated pseudogerm-free mice showed depression-like and anhedonia-like phenotype compared to control group, which were improved by FMT from mice without anhedonia.
Tillmann et al. 2019 [32]	24 adult male Flinders sensitive line (FSL) and 24 Flinders resistant line	Randomized Controlled Trial	FMT from FRL, saline, or FSL rats to FRL and FSL rats administered every third day over a 16-day period	FSL, FRL, or saline rats	OFT and FST	• Rats receiving FRL feces struggled less than saline-treated ones while there was no difference between FSL feces and saline or FSL and FRL feces.
						• Rats receiving FSL feces had significantly increased immobility compared with saline, whereas FRL feces did not differ from saline.
						• No difference in immobility between FSL and FRL feces.
Langgartner et al. 2018 [25]	Male C57BL/6 N chronically stressed mice via chronic subordinate colony (CSC)	Randomized Controlled Trial	Repeated FMT from non-stressed single-housed control (SHC) mice	Non-stressed, SHC Male C57BL/6 N mice	OFT and open-field/novel object (OF/NO) test	• SHC feces transplantation had mild stress-protective effects as shown by an improvement of CSC-induced thymus atrophy, anxiety, systemic low-grade inflammation, and alterations in bone homeostasis.
						• CSC feces transplantation slightly aggravated CSC-induced systemic low-grade inflammation and alterations in bone homeostasis in SHC and/or CSC animals.
Jiang et al. 2020 [30]	18, 7-weeek old, antibiotic treated C57BL/6 J mice	Randomized Controlled Trial	FMT from 6 control, 6 alcohol-induced depression, and 6 alcohol-induced depression nicotinamide riboside (NR) treated mice after 3 weeks of antibiotic treatment	Control, alcohol-induced depression model, and alcohol-induced depression model NR-treated C57BL/6 J mice	SPT, FST, EPM, and Y-Maze	• Mice receiving FMT from alcohol induced depression model exhibited depression-like behaviour
						• Mice receiving FMT from control or NR mice did not exhibit depression-like behaviour.
Schmidt et al. 2020 [33]	Adult female Lewis rats	Randomized Double-Blind Sham Controlled Trial	FMT from healthy rats is given to rats with spinal cord injury (SCI)	Healthy, uninjured, adult female Lewis rats	Light-dark box, Cylinder test, SPT, EPM, OFT	• FMT from healthy rats significantly reduced depression and anxiety-like behaviour resulting from SCI in the elevated plus maze and light-dark box (significantly more time in open arms of the maze and light box)
Siopi et al. 2020 [23]	8-week old, antibiotic-treated, GF C57BL/6 J mice	Randomized Controlled Trial	Antibiotic-treated GF mice receive FMT from 10 control, or 10 unpredictable chronic mild stress (USMS) mice	Control or UCMS mice	TST, FST, OFT, EPM, Light-dark Box	• FMT from UCMS mice resulted in despair-like behaviour in the TST and FST (increased immobility time in both)
Pearson-Leary et al. 2019 [24]	**Experimental Intruders:** Singly housed male Sprague–Dawley rats		FMT from vulnerable, resilient, and control rats delivered via oral gavage to naïve recipient rats once daily for 5 days.	SL/vulnerable, LL/resilient rats, or control rats	FST and SIT	• FMT from SL/vulnerable rats to naïve, non-stresses rats promotes some stress vulnerability
						• No differences in time spent interacting in SIT between recipient groups suggesting no difference in anxiety-like behavior
	**Residents:** Male Long–Evans (LE) retired breeders (600–800 g) were used as residents.					
						• Rats treated with vulnerable microbiota had increased passive depression-like behaviours (decreased latency to immobility, less time swimming, and increased time spent immobile)
						• SL/vulnerable microbiota treated rats also spent less time climbing, but this was not significant

**Table 3:** Preclinical studies with human donors

Study	Sample characteristics	Study design	Intervention	Donor	Measurement	Key findings and conclusions
De Palma et al. 2019 [43]	141 GF NIH Swiss Mice	Randomized Controlled Trial	FMT from IBS patients and healthy donors to GF mice	4 Anxious IBS-D patients, 4 non-anxious IBS-D patients, Mean age: 40 years old; 5 healthy human controls (HHC), Mean age: 42 years	**Donors:** HAM-A	• FMT from anxious IBS-patients to mice produced anxiety behaviors in mice
					**Recipients:** Light-dark preference test and step-down test	
						• FMT from IBS patients with normal anxiety and from healthy controls to GF mice showed no significant anxious behaviors in GF mice
						• Akkermansia was associated with anxiety behaviors in mice
Hata et al. 2019 [42]	Germ-free (GF) BALB/c mice	Randomized Controlled Trial	Oral FMT with and without pre-treatment with live *Bacteroides vulgatus* to GF mice	4 AN patients, Mean age: 23 years, BMI 13.7; 4 HHC, Mean age: 25.3 years, BMI 21.6	**Donors:** DSM diagnosis of AN	• FMT from AN patients induces anxiety-like and compulsive behaviors in GF recipient mice and impairs body weight gain
					**Recipients:** Open Field and Marble Burying	
						• Pre-treatment with B. vulgatus attenuates compulsive behavior
Zhao et al. 2019 [39]	Male C57BL/6 J mice with antibiotic gut microbiota suppression, 6 weeks old	Randomized Controlled Trial	FMT via intragastric administration every other day for 13 days to antibiotic treated mice	Patients with and without alcoholism, Ages 35–40	**Donors:** ICD-10 diagnosis of alcoholism	• FMT from patients with alcoholism induced spontaneous alcohol dependence in mice
					**Recipients:** Open field test (OFT), alcohol preference test (APT), elevated plus maze test (EMT), tail suspension test (TST), and social interaction test (SIT),	• FMT-Alc group exhibited anxiety-like and depression-like behaviors changes and significantly declined social interaction behaviors
Huang et al. 2019 [36]	GF Mice	Randomized Controlled Trial	FMT from MDD patients and healthy controls to GF mice	5 MDD patients; 5 HHC	**Donor:** DSM-IV diagnosis of depression and HAM-D	• FMT from MDD patients resulted in significantly increased immobility times for the FST and TST
					**Recipient:** OFT, TST, forced swimming test (FST), buried food pellet test (BFP) and olfaction behavior test (via modified BFP)	
						• The center motion distance (OFT) also significantly decreased compared to controls
						• The latency for finding the object by depressed mice was significantly longer than that by healthy controls indicating impaired olfaction.
Kelly et al. 2016 [34]	Adult, male Sprague Dawley rats treated with antibiotic cocktail for 28 days	Randomized Controlled Trial	3-day pooled sample oral FMT from MDD patients and healthy controls to antibiotic treated rats, with booster inoculations given twice per week throughout the study.	Pooled fecal samples from 3 severely depressed MDD patients; pooled fecal samples from 3 HHC	**Donor:** Perceived stress scale (PSS), Beck Depression and Beck Anxiety Scales, HAM-D, etc.	• Rats receiving FMT from MDD patients demonstrated anhedonia-like behaviours as shown by a significant decrease in sucrose intake without affecting fluid intake in SPT
					**Recipients:** Sucrose preference test (SPT), OFT, EMT, FST	
						• Rats receiving FMT from MDD patients also exhibited anxiety-like behaviours as shown by a significant decrease in visits to the open arms in the EMT and a reduction in time spent in the centre in the OFT.
						• In the forced swim test, there were no significant differences between the groups in immobility time, swimming, or climbing.
Xu et al. 2018 [40]	110 male C57BL/6 mice aged 4 to 5 weeks exposed to chronic ethanol	Randomized Controlled Trial	**FMT 1:** FMT at the end of chronic alcohol exposure period	3 HHC	**Recipients:** OFT, TST, FST, and APT	• FMT 1 could not alleviate alcohol-induced anxiety or depression.
			**FMT 2:** FMT at middle (6%) of alcohol exposure period.			• FMT 2 alleviated alcohol-induced depression in TST
			**FMT 3:** FMT at the beginning of whole exposure period			• FMT 3 modulated anxiety and significantly improved depression.
						• FMT 3 decreased Anxiety in OFT and significantly improved depression in TST.
						• No significant alcohol preference alternation in FMT-treated mice.
Zheng et al. 2016 [35]	Male GF Kunming mice and specific pathogen free (SPF) Kunming mice, 6–8 weeks old	Randomized Controlled Trial	Pooled sample FMT from MDD patients and HHC to GF mice	5 Male MDD patients, ages 26–61; 5 Male HHC, ages 29–6	**Recipients:** OFT, FST, TST	• Absence of gut microbiota in germ-free (GF) mice resulted in decreased immobility time in the FST compared to conventionally raised HC mice
						• The gut microbiota compositions of MDD patients and HC were significantly different with MDD patients characterized by significant changes in the relative abundance of Firmicutes, Actinobacteria and Bacteroidetes.
						• FMT from MDD patients to GF mice resulted in depression-like behaviors compared to HC colonization
						• Weight was not significantly different between groups
Chen et al. 2020 [69]	ASD Model Mice	Randomized Controlled Trial	Pooled samples from Healthy Human donor gut bactiera (M + O) or cultured bacteria from original pooled healthy donor gut bacteria (M+ F)	Original healthy Human bacteria (M + O); in vitro cultured bacteria from healthy human donor (M + F)	OFT, Marble Burying Test, Self-grooming, Three-Chamber Social Test	• M + O spent significantly more time and had more entries in the OFT, significantly lower % of marbles burried, and significantly lower % of gromming time.
						• M + F had significantly lower % of marbles burried, and % of grooming time.
						• These results suggest that FMT from organic in vivo microbiota may be better at alleviating depressive and anxiety-like behaviours, but that both in vivo snd in vitro bacteria transplantation have beneficial properties.
Liu et al. 2020 [37]	18, 8-week old GF rats		Microbiota from healthy or depressed humans were transplanted into GF mice	Depressed or Healthy Humans between ages 18–60	**Donors:** 24+ points on HAM-D, DSM-5 diagnosis of MDD**Recipients:** FST, SPT	• Rats receiving depression microbiota exhibited depression-like behavior (immobility time in the forced swimming test was significantly higher than in control groups)
						• From the first week to the fourth week, the saccharine preference index was significantly lower in the depression micro- biota group than that in the blank control and healthy microbiota groups

**Table 4:** Clinical studies

Study	Sample characteristics	Study design	Intervention	Donor	Measurement	Key findings and conclusions
Cai et al. 2019 [66]	1 female MDD patient, 79 years old	Pre- and post-intervention assessment	Single time FMT via gastroscope	6 year-old grandson	PHQ-9	• Six months after intervention PHQ-9 scores improved.
						• Significant increase in Firmicutes counts and Bacteroides significant reduced
De Clerq et al. 2019 [67]	1 female AN patient, 26 years old	Case report, pre- and post-intervention assessment	Single duodenal FMT	Unrelated female donor with BMI of 25	BMI, caloric intake	• Increase in BMI post-intervention
						• No significant differences in gut microbiota composition after FMT
Huang et al. 2019 [45]	30 (18 M, 12F) refractory IBS patients, Mean age: 44 years old	Pre- and post intervention assessment with 1, 3, and 6 month follow-ups	Two to three FMT procedures (done every other day) via colonoscopy	Healthy volunteers aged 8–35	IBS-QOL, IBS-SSS, GSRS, HAM-A and HAM-D	• Significantly improved GI symptoms and alleviated depression and anxiety as indicated by IBS-QOL, IBS-SSS, GSRS, HAM-A and HAM-D scores, 1 and 3 months post-FMT
						• Increase in Verrucomincrobia and Euryarchaeota at phyla level and increase in Methanobrevibacter and Akkermansia at the genus level, at 1 month after FMT compared to before FMT
Mazzawi et al. [46]	13 (9 M, 4F) IBS patients, Mean age: 32 years old	Open label, pilot study	Single duodenal FMT via gastroscope	Healthy donors, aged 20–42	IBS-SQ, IBS-SSS, EPQ-N-12, and HAD	• Scores of all questionnaires improved significantly at all follow-up time points and lasted up to 28 weeks
						• Patients’ microbiota compositions became more similar to donors after FMT
Mizuno et al. 2017 [44]	10 (7 M, 3F) refractory IBS patients, Mean age: 40.1 years old	Single arm, open label, non-randomized study with 12-week follow-up	Single time FMT via colonoscopy	Healthy relatives in second-degree relationship, Mean age: 52 years	HAM-A, HAM-D	• The HAM-D score significantly improved 4 weeks after FMT but returned to the baseline level at 12 weeks
						• When evaluated with HAM-A, the GI symptoms significantly improved from before FMT to 12 weeks after in responders, but not in non-responders
						• Significant increase in microbial diversity from before treatment to week 4
						• Significant relationship between diversity and response to treatment at week 4 but not before treatment
Xie et al. 2019 [47]	1 male MDD patient with alopecia and GI symptoms, 86 years old	Case report, pre- and post-intervention assessment	Six rounds of FMT via colonoscopy	22-year old healthy male donor	HAM-D	• Improved depressive symptoms
						• Improved appetite and no abdominal pain or distension, increased BMI.
						• Improved hair growth without any hair loss treatments
Kurokawa et al. 2018 [48]	17 (8 M, 9F) IBS patients, Mean age: 43.41	Single arm, non-randomized, open label, observational study	Single time FMT, via colonoscopy	Healthy relatives in second-degree relationship, Mean age: 51.41 years	HAM-D and subscale of sleep-related items, HAM-A, and QIDS	• Significant improvement in HAM-D total and sleep subscale score, HAM-A, and QIDS after FMT, at times even without GI symptoms improvement
						• Significant increase in microbiome diversity after FMT
Johnsen et al. 2020 [49]	85 IBS (non-constipated) patients between 18 and 75 years of age	Double-blind, Randomized Controlled Trial, Parallel group	FMT (frozen or fresh) using health donors or using patient’s own feces, delivered to cecum of IBS patients via colonoscope	Frozen or fresh feces from healthy donors	Fatigue Impact Scale (FIS), IBS-QoL, IBS-SSS	• Clinical effect on QoL and fatigue six months after treatment, with waning effect from six to twelve months,
						• Transient treatmnet effect seen in individuals with other functional disorders.
						• Absence of other self reported functional disorders and presence of depression at baseline is suggested to predict a lasting effect of FMT in QoL and fatigue, respectively

## Abbreviations

GBA: Gut-Brain Axis
GI: Gastrointestinal
CNS: Central Nervous System
ANS: Autonomic Nervous System
IBS: Irritable Bowel Syndrome
BDNF: Brain-Derived Neurotrophic Factor
FMT: Fecal Microbiota Transplant
GAD: Generalized Anxiety Disorder
MDD: Major Depressive Disorder
PRISMA: Preferred Reporting Items for Systematic Reviews and Meta-Analyses
CUMS: Chronic Unpredictable Mild Stress
NLRP3: NOD-, LRR- and pyrin domain-containing protein 3
CSC: Chronic Subordinate Colony
SHC: Non-stressful Single Housing Colony
FSL: Flinders Sensitive Line Rats
FRL: Flinders Resistant Line Rats
LL/Resilient: Long Latency/Resilient
SCI: Spinal Cord Injury
SNI: Spinal Neuropathic Injury
SL/Vulnerable: Short Latency/Vulnerable
GF: Germ Free
HAM-D: Hamilton Depression Rating Scale
PHQ-9: Patient Health Questionnaire-9
QIDS: Quick Inventory of Depressive Symptomatology
HADS(−A/D): Hospital Anxiety and Depression Scale (−Anxiety/Depression Specific)
HAM-A: Hamilton Anxiety Rating Scale
EPQ-N-12: Eynsek Personality Questionnaire-Neuroticism
IBS-QOL: Irritable Bowel Syndrome – Quality of Life
SSRI: Selective Serotonin Reuptake Inhibitors
EC: Enterochromaffin Cells
SCFA: Short-chain Fatty Acids
